# Genetic mapping of a new race specific resistance allele effective to *Puccinia hordei* at the *Rph9/Rph12* locus on chromosome 5HL in barley

**DOI:** 10.1186/s12870-014-0382-4

**Published:** 2014-12-20

**Authors:** Peter M Dracatos, Mehar S Khatkar, Davinder Singh, Robert F Park

**Affiliations:** The University of Sydney, Plant Breeding Institute Cobbitty, Private Bag 4011, Narellan, 2567 NSW Australia; Faculty of Veterinary Science, University of Sydney, 425 Werombi Road, Camden, 2570 NSW Australia

**Keywords:** Resistance, *Puccinia hordei*, Genetic mapping

## Abstract

**Background:**

Barley is an important cereal crop cultivated for malt and ruminant feed and in certain regions it is used for human consumption. It is vulnerable to numerous foliar diseases including barley leaf rust caused by the pathogen *Puccinia hordei*.

**Results:**

A temporarily designated resistance locus *RphCantala* (*RphC*) identified in the Australian *Hordeum vulgare* L. cultivar ‘Cantala’ displayed an intermediate to low infection type (“;12 = N”) against the *P. hordei* pathotype 253P- (virulent on *Rph1, Rph2, Rph4, Rph6, Rph8* and *RphQ)*. Phenotypic assessment of a ‘CI 9214’ (susceptible) x ‘Stirling’ (*RphC*) (CI 9214/Stirling) doubled haploid (DH) population at the seedling stage using *P. hordei* pathotype 253P-, confirmed that *RphC* was monogenically inherited. Marker-trait association analysis of *RphC* in the CI 9214/Stirling DH population using 4,500 DArT-seq markers identified a highly significant (−log_10_Pvalue > 17) single peak on the long arm of chromosome 5H (5HL). Further tests of allelism determined that *RphC* was genetically independent of *Rph3, Rph7*, *Rph11, Rph13* and *Rph14,* and was an allele of *Rph12* (*Rph9.z*), which also maps to 5HL.

**Conclusion:**

Multipathotype tests and subsequent pedigree analysis determined that 14 related Australian barley varieties (including ‘Stirling’ and ‘Cantala’) carry *RphC* and that the likely source of this resistance is via a Czechoslovakian landrace LV-Kvasice-NA-Morave transferred through common ancestral cultivars ‘Hanna’ and ‘Abed Binder’. *RphC* is an allele of *Rph12* (*Rph9.z*) and is therefore designated *Rph9.am*. Bioinformatic analysis using sequence arrays from DArT-seq markers in linkage disequilibrium with *Rph9.am* identified possible candidates for further gene cloning efforts and marker development at the *Rph9*/*Rph12*/*Rph9.am* locus.

**Electronic supplementary material:**

The online version of this article (doi:10.1186/s12870-014-0382-4) contains supplementary material, which is available to authorized users.

## Background

Leaf rust, caused by *Puccinia hordei*, is one of the most destructive foliar diseases of barley, and has caused significant yield losses in many regions where barley is grown [1-3]. Yield reductions of up to 32% have been reported in certain susceptible barley cultivars in both Australia and North America [4]. Due to potentially adverse environmental effects of fungicides, the most preferable and cost-effective means of controlling barley leaf rust is through the development and deployment of durable host resistance [5].

In cereals, two major types of resistance have been described for rust pathogens, seedling resistance and adult plant resistance (APR). Seedling resistance genes are effective at all stages of crop development and are often characterized by a hypersensitive response. Numerous genes conferring seedling resistance to *P. hordei* (*Rph*) have been identified (*Rph1-Rph19* [6], *Rph21-Rph22* [7,8]), however, virulence matching most of these genes has been detected [9]. In some regions, including South Australia, the presence of the alternate host *Ornithogalum umbellatum* (‘Star of Bethlehem’) can permit sexual recombination and increase the likelihood of new virulent pathotypes developing [9-12]. New sources of seedling resistance are required for use in breeding programs in combination with APR for durable protection against *P. hordei*. Furthermore, for effective deployment within breeding programs, it is equally important to understand the mechanisms of inheritance and pathotype specificity of newly identified resistance genes.

Previous studies on the inheritance of seedling resistance to *P. hordei* have determined that many of the known *Rph* loci are complex. From a total of 23 catalogued *Rph* genes, three have been previously reported to be alleles of other genes. *Rph5* is allelic to *Rph6* [13], *Rph12* is allelic to *Rph9* [14] and *Rph15* is allelic to *Rph16* [15]. In the case of *Rph9* and *Rph12*, a large F_2_ population of 3,858 progeny derived from ‘HOR 2596’ (*Rph9*) x ‘Triumph’ (*Rph12*) was evaluated and no recombinants were detected, suggesting that both are alleles of the same gene [14]. *Rph9* and *Rph12* also mapped to the same locus on chromosome 5H and were linked to a common molecular marker, ABC155. Previous studies have determined that the Australian cultivar ‘Cantala’ carries an uncharacterised seedling gene for resistance to *P. hordei* that was temporarily designated *RphCantala* (*RphC*) [1]. Recent evidence suggests the *RphC* is present in several Australian and European barley cultivars and was originally derived from European descent. Although virulence for *RphC* is common among Australian populations of *P. hordei*, such resistance may be useful in combination with other resistance sources. This study reports on the characterization and genetic mapping of the *RphC* resistance. Data on both the physical location and possible candidate genes for the *RphC* resistance locus are presented and discussed.

## Methods

### Plant and pathogen material

A doubled haploid (DH) population, CI 9214/Stirling, derived from ‘CI 9214’ (PI 186125) (postulated to carry *Rph1*; R. F. Park, unpublished) and ‘Stirling’ (PI 466919) (*RphCantala*; [1], R. F. Park, unpublished) with 258 progeny was used for genetic analysis in this study. F_3_ populations derived by intercrossing ‘Cantala’ (PI 483047) with ‘Estate’ (*Rph3*) (CI 3410), ‘Cebada Capa’ (CI 6193) (*Rph7*), ‘Clipper BC8’ (*Rph10*)*,* ‘Triumph’ (*Rph12*) (PI 186125), and *‘*PI 531849’ (*Rph13*) were used for tests of allelism. A total of five pathotypes of *P. hordei* used in the study along with their virulence/avirulence profiles and reactions to barley differential lines and Australian cultivars postulated to carry *RphC* are listed in Table 1. All pathotypes used originated from annual pathogenicity surveys of *P. hordei* conducted in Australia and are maintained in liquid nitrogen at the Plant Breeding Institute, University of Sydney.

**Table 1:** Seedling response of selected barley genotypes to five Australian pathotypes of *Puccinia hordei*

		**Pathotype [accession number** ^**1**^ **]**				
**Cultivar**	**Resistance gene**	**200P-** ^**2**^ **[S3088** ^**3**^ **]**	**200P+ [900233]**	**243P- [920636]**	**253P- [760462]**	**4610P+ [900380]**
Sudan	*Rph1*	;1 N	;1 N	3+	3+	;1 N
Peruvian	*Rph2*	;12 = C	;12 = CN	3+	33+	;12 = CN
Estate	*Rph3*	;	;	;	;	;
Gold	*Rph4*	;12-	;1 + N	;12-	3+	3+
Magnif 104	*Rph5*	;N	;1 N	;N	;N	;N
Bolivia	*Rph2* + *Rph6*	;12 = C	;12 = CN	33 + C	33+	;12 = CN
Cebada Capa	*Rph7*	0;N	0;N	0;N	0;N	0;N
Egypt 4	*Rph8*	33+	33+	3+	3+	3+
Abyssinian	*Rph9*	;12 = C	;2 = C	;12 = C	;1-CN	3+
Clipper	*-*	3+	3+	3+	3+	3+
Clipper BC8	*Rph10*	3+	;1CN	;1 + N	2++3	2++3
Clipper BC67	*Rph11*	12-N	12+	22+	22-C	2-C
Triumph	*Rph12*	12 = N	12 = N	22+	12 = N	3+
PI 531849	*Rph13*	;N	;-N	;N	;N	;N
PI 584760	*Rph14*	;12-C	;12-C	2+	2+	22 + C
Bowman*4/PI355447	*Rph15*	;N	;N	;N	;N	;N
Q21861	*RphQ*	;1 N	;1 N	3+	3+	;12 = N
38P18^4^	*Rph18*	;-N	;-N	;-N	;-N	;-N
Reka 1	*Rph2* + *Rph19*	;N	;1 + CN	;12-N	;1 N	;12 = N
Prior	*Rph19*	;1 N	3+	;1-N	;N	3+
Ricardo	*Rph2* + *Rph21*	;12-N	;12-N	2 + 2++	2++	12 = N
Cantala	*RphCantala*	3+	3+	3+	;12 = CN	3+
Bandulla	*RphCantala*	3+	3+	3+	;12 = CN	3+
Bussell	*RphCantala*	3+	3+	3+	;12 = N	3+
Chebec	*RphCantala* + *Rph19*	12-C	3+	12-CN	;12 = CN	3+
Hannan	*RphCantala*	3+	3+	3+	;12 = CN	3+
Lara	*RphCantala*	3+	3+	3+	;12 = N	3+
Milby	*RphCantala*	3+	3+	3+	;12 = N	3+
Moondyne	*RphCantala*	3+	3+	3+	;12 = N	3+
Noyep	*RphCantala*	3+	3+	3+	;12 = N	3+
Parwan	*RphCantala*	3+	3+	3+	;12 = N	3+
Research	*RphCantala*	3+	3+	3+	;12 = N	3+
Resibee	*RphCantala*	3+	3+	3+	;12 = N	3+
Tilga	*RphCantala*	3+	3+	3+	;12 = N, 3+	3+
Stirling	*RphCantala*	3+	3+	3+	;12 = N	3+

^1^Plant Breeding Institute Cobbitty rust collection accession numbers.

^2^P- and P+ indicate avirulence and virulence, respectively, for *Rph19.*

^3^Culture kindly provided by Dr R.G. Rees, Queensland Department of Primary Industries.

^4^Original seed kindly supplied by Dr R.A. Pickering, New Zealand Institute for Crop and Food Research Limited.

### Sowing, inoculation and disease assessment procedures

Sowing and inoculations were performed as described by Sandhu et al. [6]. Disease response was assessed 12 days after inoculation using a modified “0” – “4” scale as described by McIntosh et al. [16]. Variations of the infection types were indicated by the use of “-” (less than average for the class), ‘+’ (more than average for the class), ‘C’ (chlorosis), ‘N’ (necrosis) and “X” which denotes a mesothetic infection type with a mixture of infection types on the same leaf. A comma separating different infection types was used to indicate heterogeneity within a given test host genotype. When two different infection types were observed on a single leaf, they were written together without a comma.

### Genetic mapping of *RphC* in the CI 9214/Stirling DH population

Genomic DNA was extracted from the leaf tissues of a single plant from a subset of 61 from the 258 original CI 9214/Stirling DH lines using CTAB essentially as described by Fulton et al. [17]. The DNA of each DH line was diluted to 100 ngμL^−1^ and subjected to genotypic analysis using the DArT-seq platform essentially as described by Curtois et al. [18], except that the marker curation involved removing the markers with low minor allelic frequency (MAF) (*i.e.* < 0.1) and > 50% missing data.

Genetic linkage maps were constructed using MSTMap software [19]. The following specific parameters of MSTMap were used *viz.* name for the mapping population: DH; the distance function: Kosambi; the threshold to be used for clustering the markers into LGs: 0.000001; the objective function: COUNT. In addition, any group of markers less than two and with a distance of 15 centimorgans (cM) away from the rest of the markers was placed in a separate linkage group. This linkage map of the CI 9214/Stirling DH population was specifically constructed for genetic mapping of *RphC*, for this the phenotypic data of *P. hordei* pathotype 253P- was converted to binary data [(susceptible 3+ =0 or resistant i.e. ;12 = CN” =1) and was then included as an additional marker. The map positions (cM) of all closely linked DArT-seq markers to *RphC* on the CI 9214/Stirling genetic map were compared with the Bowman consensus map and the *Hordeum vulgare* L. cv. ‘Bowman’ genome assembly [20].

### Marker-trait and bioinformatic analysis of closely linked DArT markers at the *RphC* locus

Marker-trait analysis of each DArT marker with the *RphC* phenotype was conducted by computing Fisher’s exact test on 2 X 2 count tables using R statistical software (www.r-project.org). The null hypothesis was that the DArT marker genotypes were not associated with resistance to *P. hordei*; hence a random distribution of genotypes in the resistant and susceptible phenotypic groups. The –log10 of P values were plotted against the positions on the physical Bowman genome assembly [20] by means of chromosome-wise and genome-wide ‘Manhattan’ plots.

Linkage disequilibrium (LD) *r*^*2*^ was measured between the binary scores of the *RphC* phenotype (0, 1) with each DArT-seq marker genotype using GOLD [21]. The correlation coefficient of each marker with *RphC* binary phenotypic score was plotted against the Bowman genome assembly by means of a genome-wide ‘Manhattan’ plot. The sequences of DArT-seq markers with *r*^*2*^ > 0.8 were individually blasted (blastn) against the ‘Morex’ barley genome sequence browser (www.gramene.org) to identify the physical scaffold of genes in the region between markers flanking *RphC* based on relationships between the CI 9214/Stirling DH population genetic map and the 'Bowman' consensus genetic maps. The physical positions and annotations of all genes located between DArT-seq marker DART461 (504808312–504808380) and DART4872 (509749584–509749620) were tabulated to identify possible candidates for *RphC*. Further Pfam protein annotations were also assigned to the DArT-seq markers that were located within predicted genes in the ‘Morex’ genome. The haplotype blocks in the significant region were constructed using Haploview [22] to examine the LD in the region and among significant DArT-seq markers.

## Results

### Multipathotype tests

The barley cultivar ‘Cantala’ contained an uncharacterised seedling resistance gene (*RphC*) to *P. hordei* that was identified through phenotypic assessment of the Australian barley differential lines (including ‘Cantala’) with a range of *P. hordei* pathotypes (Table 1). Multi-pathotyping tests on other barley cultivars suggested that that in addition to ‘Cantala’ , ‘Bandulla’ , ‘Bussell’ , ‘Chebec’ (with *Rph19*), ‘Hamelin’ , ‘Lara’ , ‘Milby’ , ‘Moondyne’ , ‘Noyep’ , ‘Parwan’ , ‘Research’ , ‘Resibee’ , ‘Tilga’ (heterogeneous) and ‘Stirling’ also carry *RphC*. None of these cultivars carried *Rph12* (Table 1).

### Genetic analyses of DH population CI 9214/Stirling

**Figure 1:**
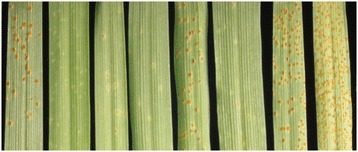
**Seedling leaves of the infection types of (L to R): (1) CI9214 (** ***P. hordei*** **pathotype [pt] 253P-/Virulent** ***Rph1*** **) (2) CI9214 (pt 210P+/avr** ***Rph1*** **) (3) Stirling (pt 253P-/avr** ***RphCantala*** **) (4) Cantala (pt 253P-/avr** ***RphCantala*** **) (5) Cantala (pt 210P+/vir** ***RphCantala*** **) (6) Triumph (pt 253P-/avr** ***Rph12*** **) (7) Gus (pt 253P-/vir) and (8) Gus (pt 210P+/vir).**

The parental genotypes of the CI 9214/Stirling DH population were postulated to carry *Rph1* and *RphC* based on the observed infection types (IT) in response to *P. hordei* pathotypes 200P- (avirulent for *Rph1* and virulent for *RphC*) and 253P- (virulent for *Rph1* and avirulent for *RphC*), respectively (Figure 1; Table 1). Pathotype 200P- was avirulent on ‘CI 9214’ (*Rph1*) and ‘Triumph’ (*Rph12*) and virulent on ‘Cantala’ (*RphC*). Conversely, pathotype 253P- was virulent on ‘CI 9214’ (*Rph1*) and avirulent on ‘Stirling’ (*RphC*), ‘Cantala’ (*RphC*) and ‘Triumph’ (*Rph12*). Both pathotypes were virulent on the universal susceptible line ‘Gus’ (Figure 1; Table 1). The CI 9214/Stirling population of 258 DH lines was phenotyped using pathotype 253P- and in response the resistant parent ‘Stirling’ gave the same IT “;12 = CN” as observed in the barley variety ‘Cantala’ with the same pathotype, whilst the susceptible parent ‘CI 9214’ gave IT “3+” to this pathotype. The observed segregation within the CI 9214/Stirling population fitted with a predicted single gene inheritance model 1:1 ratio using Chi squared analysis (121 resistant: 137 susceptible (P > 0.3)).

### Genetic mapping of *RphC*

**Figure 2:**
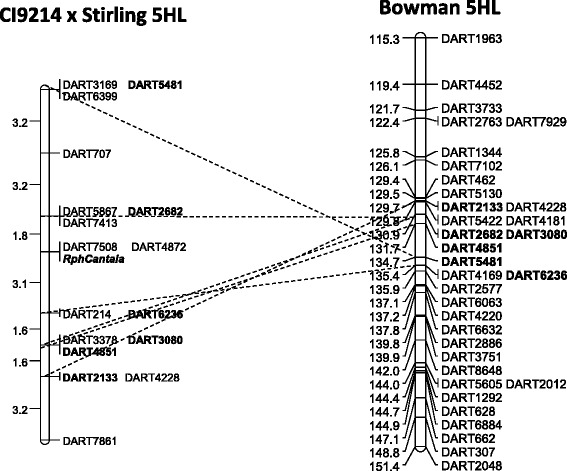
**Partial linkage maps of linkage group seven of nine of the CI9214/Stirling doubled haploid population encompassing leaf rust resistance gene** ***RphCantala*** **.** Comparative map analysis was performed using common DArT markers between the CI9214/Stirling and the 'Bowman' consensus DArT-seq genetic map. DArT markers in common are in bold.

**Figure 3:**
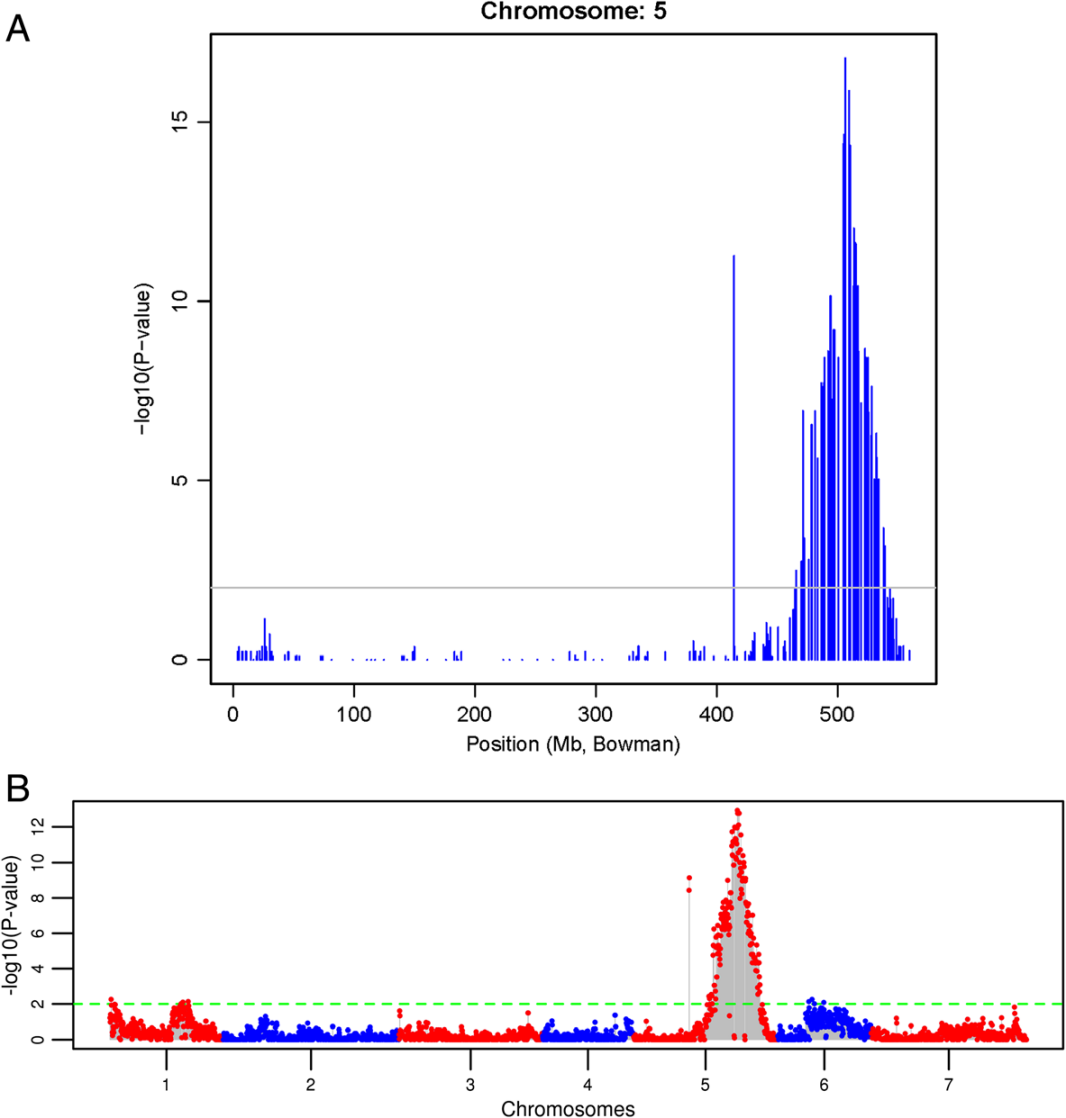
**Marker trait association analysis scans using Fisher’s exact test Vertical axis represents -log10 (P) values of the P-value of the marker trait association.** The peaks above minimum threshold of 2 (P-value = 0.03) can be considered as significantly associated. The colours blue and red were used to differentiate between chromosomes (1H-7H). **(A)** Chromosome-wise plot and **(B)** genome-wide manhattan plot of chromosome 5HL derived from marker-trait association (MTA) analysis using Fisher’s exact test on 2 X 2 count table for seedling resistance to *Puccinia hordei* pathotype 253P- (binary scoring data) in the CI9214/Stirling doubled haploid population using 4, 500 DArT-seq markers. The –log10 of P-values were plotted against the positions on the physical Bowman genome assembly [20]. The peaks above minimum threshold of 2 (P-value = 0.03) can be considered as significantly associated.

A total of 61 representative genotypes of the CI 9214/Stirling DH population from both resistant and susceptible phenotypic classes were selected for genetic mapping of *RphC* and subsequently genotyped using 10,258 DArT-seq marker loci. A genetic map was constructed and contained nine linkage groups spanning 4,246 cM using over 4,500 DArT-seq markers, which include the *RphC* binary phenotype as a marker. Based on the known positions of flanking markers on the consensus ‘Bowman’ , ‘Morex’ and ‘Barke’ genetic maps, *RphC* was mapped to chromosome 5HL between 129–134 cM (Figure 2). *RphC* co-segregated with two DArT markers (DART4872 and DART7508) and was 1.8 cM distal to the flanking markers DART2682, DART5867 and DART7413 and 3.9 cM proximal to DART6236 and DART214 (Figure 2). Further genome-wide marker-trait association demonstrated that DArT sequences only on 5HL were associated with *RphC* phenotypic scores indicated by two significant peaks [−log10(P-value) of 17.5], across the entire genome, at approximately 506 Mb on 5HL (Figures 3A and 3B) and correlated with LD mapping results of *RphC* (Additional file 1). Further linkage disequilibrium analysis identified that the 2^nd^ peak at 430 Mb was due to incorrect map position of a single DArT marker (data not shown).

### Tests of allelism

Tests of allelism between *RphC* and two previously identified *Rph* seedling genes on chromosome 5HL (*Rph12* and *Rph13*) indicated that *RphC* is independent of *Rph13* but completely linked with *Rph12*. A two-gene segregation (fitting 7Res: 8Seg: 1Sus model) was observed in F_3_ families involving crosses of *RphC* with *Rph13* when tested with pathotype 253P- (Table 2). On other hand, there was no segregation among F_3_ families of cross involving *RphC* and *Rph12* when tested with pathotype 253P- (avirulent for both *RphC* and *Rph12*). This suggests that *RphC* is an allele of *Rph12* (*Rph9.z*) with distinct specificity and can therefore be given the allele designation *Rph9.am*. Two additional populations derived from ‘Estate’ (*Rph3*)/*‘*Cantala’ and ‘Cebada Capa’ (*Rph7*)/‘Cantala’ were also tested with pathotype 253P- and both populations conformed to expected two gene segregation model (Table 2).

**Table 2:** Chi squared analysis of barley populations for tests for allelism with *RphCantala*

**Population**	**Genes involved**	**Pop**	**No. of F**_**3**_ **families**			**Genetic ratio**	**P value**	**Chi square**
			**NSR**	**Seg**	**NSS**			
Cantala/Triumph	*RphC/Rph12*	F_3_	208			No segregation^#^	<0.0001	267.429^a^
Cantala/PI531849	*RphC/Rph13*	F_3_	69	71	10	7:8:1	0.807	0.429^a^
Cantala/Estate	*RphC/Rph3*	F_3_	112	87	18	7:8:1	0.012	8.779^a^
Cantala/Cebada Capa	*RphC/Rph7*	F_3_	72	107	8	7:8:1	0.117	4.290^a^
Stirling/CI9214	*RphC/Rph1*	DH	121		137	1:1	0.319	0.992^b^

Maximum recombination r = 1.1 cM (P = 0.01) and r = 0.7 cM (P = 0.05) calculated from Hanson [32] based on the hypothesis that the two loci are different.

^#^Chi squared values are denoted a and b for 2 and 1 degrees of freedom, respectively.

### Pedigree analysis for *RphC* resistance

**Figure 4:**
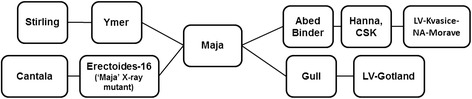
**Pedigree relationship of barley varieties ‘Stirling’ (PI 466919) and ‘Cantala’ (PI 483047/AUS 99074) (postulated to carry** ***RphCantala*** **based on multipathotype tests and genetic mapping analysis) tracing the** ***RphC*** **resistance back to Swedish and Czechoslovakian landraces LV-Gotland and LV-Kvasice-NA-Morave.** ‘Cantala’ is derived from pedigrees ‘Kenia’ and ‘Erectoides 16’, a mutant derived from the Danish cultivar ‘Maja’.

Pedigree analysis was performed on all 14 Australian barley cultivars postulated to carry *RphC* including: ‘Bandulla’ , ‘Bussell’ , ‘Cantala’ , ‘Chebec’ , ‘Hamelin’ , ‘Lara’ , ‘Milby’ , ‘Moondyne’ , ‘Noyep’ , ‘Parwan’ , ‘Research’ , ‘Resibee’ , ‘Tilga’ (heterogeneous) and ‘Stirling’ using the online barley pedigree resource http://genbank.vurv.cz/barley/pedigree/. On this basis, cultivars ‘Gull’ and ‘Binder’ were predicted to be the sources of the *RphC* resistance in ‘Stirling’ , ‘Bandulla’ , ‘Noyep’ , ‘Lara’ , ‘Stirling’ , ‘Research’ , ‘Chebec’ , ‘Moondyne’ , ‘Bussell’ and ‘Cantala’. Pedigree analysis suggests that ‘Maja’ (pedigree of ‘Ymer’ and ‘Erectoides 16’) share common ancestry of ‘Binder’ and ‘Gull’ (Figure 4). Both ‘Gull’ and ‘Binder’ were derived from landraces from Sweden (‘LV-Gotland’) and ‘Czechoslovakia’ (LV-Kvasice-NA-Morave through ‘Hanna’), respectively (Figure 4). The donor sources of seedling resistance in ‘Hannan’ , ‘Milby’ and ‘Tilga’ could not be explained based on available pedigree information.

### LD and bioinformatic analysis of closely linked DArT markers at the *RphC* locus

A total of 15 DArT-seq markers had an *r*^*2*^ > 0.8 and five of these were in complete LD (*r*^*2*^ = 1) with *RphC* (Table 3; Figure 3; Additional file 1). Table 4 provides a list of DArT locus name, clone ID and associated sequences for each marker. DArT-seq markers DART4872 and DART7508 that co-located with *RphC* in the CI 9214/Stirling genetic map were both in complete LD with *RphC*, however, they along with DART7846 were not present in the ‘Bowman’ consensus maps (Table 3; Figure 2). Furthermore, the sequence of the most closely associated DArT-seq marker based on the lowest Fisher's exact test P value to *RphC* (DArT4851) was located within a predicted disease resistance protein (NB-ARC) based on Pfam analysis (Table 3). Two other sequences (DART7846 and DART3079) were located within the same transcript of another predicted disease resistance gene (serine/threonine receptor kinase gene) on chromosome 5HL. In the ‘Morex’ genome both DART7846 and DART3079 had a closest match to a physical position 9520434–9520483 distant to all other closely associated DArT sequences, but the contig in the ‘Bowman’ assembly mapped to physical position 506583400 (Table 3)

**Table 3:** Details of bioinformatic analyses of DArT-seq markers closely linked to *RphCantala*

**DArT locus_name**	**r** ^**2**^ **with** ***RphCantala***	**Bowman cM**	**C/S cM**	**Fisher's P-value**	**Barley physical**	**Bowman physical**	**Blastn P-value**	**Morex physical**	**Pfam Morex 5HL**
DART4851	1	131.67	97.30	1.69E-17	5HL	506583400	3.40E-27	506781405-506781469	NB-ARC
DART4872	1	NA	90.74	1.69E-17	5HL	NA	1.80E-11	509749584-509749620	No match
DART7507	1	NA	97.30	5.33E-16	5HL	NA	2.00E-16	506713993-506714061	Tubulin -GTPase
DART7846	1	NA	NA	3.93E-12	5HL	NA	1.30E-19	9520434-9520483	Serine/threonine Protein Kinase
DART3079	0.937	130.9	102.04	1.38E-16	5HL	506583400	5.80E-32	9519766-9519834	Serine/threonine Protein Kinase
DART3263	0.937	134.72	NA	1.38E-16	5HL	509518480	1.40E-29	509705303-509705371	No match
DART5481	0.918	134.72	89.13	1.05E-12	5HL	509518480	5.80E-32	506948877-506948945	Inosine-5′-monophosphate dehydrogenase
DART2133	0.875	129.72	103.65	4.13E-15	5HL	505380600	1.40E-29	505484624-505484692	No match
DART2681	0.873	130.9	102.04	4.13E-15	5HL	506583400	1.40E-29	506600697-506604765	No match
DART6198	0.873	129.44	110.31	4.13E-15	5HL	504740440	1.40E-29	504879962-504880030	Cytochrome P450
DART4228	0.867	129.72	103.64	1.52E-14	5HL	505380600	5.80E-32	505484082-505484150	No match
DART4182	0.867	129.83	110.307	5.60E-14	5HL	505380600	5.80E-32	505506713-505506781	AP2 transcription factor
DART5422	0.857	129.83	111.92	7.50E-13	5HL	505380600	1.40E-29	505507578-505507646	AP2 transcription factor
DART461	0.817	129.44	115.15	6.41E-14	5HL	504780440	5.80E-32	504808312-504808380	Uncharacterised protein

NA, not available.

**Table 4:** Summary information for significant DArT-seq markers in linkage disquilibrium (r^2^ > 0.8) with *RphCantala* on chromosome 5HL

**Locus name**	**DArT clone ID**	**Sequence**
**DART4872**	100025017|F|0-28:G > C-28:G > C	TGCAGCAAAAATAGCACCGCCACACAACGTGCGCGGCAGCGCTCCCTCCAGCGACGCGACGCCTAGGAT
**DART7508**	100020485|F|0-40:G > A-40:G > A	TGCAGGGGGCAAGAGCAAACAAGGCATGATGAGCAAACCAGGCATGGTTGAGAGATCAGGCTAATTGTT
**DART7846**	100023795|F|0-13:G > A-13:G > A	TGCAGTACCTCGCGCTCTCCGGCAACGAGCTGTCTGGCAAGATACCGCCGAGATCGGAAGAGCGGTTCA
**DART3080**	100017949|F|0-37:A > G-37:A > G	TGCAGCTCGAAGAGACCCTTGGGAATGGAGCCGTTCAAGTAGTTCTCTCCGAGACGGATACGGCTCAAG
**DART3263**	100023711|F|0--33:C > A	TGCAGGCTCCCGCAGCCGCTGCTCCGTTCATCCCCCAGCAGCACCAGTACGTCACTCAGACGGGCACGG
**DART5481**	100009066|F|0--42:A > G	TGCAGCCAACCTTGGATGGAACACACAGAAAACCAGTATGCCAGTCCTCGATTTAAAGATGGGGCTAGA
**DART2133**	100025595|F|0--17:C > A	TGCAGTCCTGCTCATCTCTTCTTATGGTGACTACATGTTCTTCTTCATCTGGCTGGTCTGAGTTGGATG
**DART2681**	100009568|F|0--11:A > G	TGCAGTGGAATATAGCAAGGGCGGAGCAGCACAGTCAGTCAAGTCTGCATGCATGCGAGCAACCGTTGC
**DART6198**	100012213|F|0-56:T > C-56:T > C	TGCAGGTTTTTGAACTTTGAAGAACTCGCGCCGCTGCCTTGGGAAAATGTTTGAATTGTCCAAGGACAT
**DART4228**	100017110|F|0-68:A > G-68:A > G	TGCAGTTAGTCCAAGAAAGAGGAAAGCTGATGATGGTCTCAGTCTCAGTCCAAGAAAGAGGAAAGCTGA
**DART4182**	100011760|F|0-59:G > A-59:G > A	TGCAGATTTGTAGTCCACTAGGTACTAGTACTATCTTGTAGCGAGATTGCGAGGTTGCAGTCTCAGGGA
**DART5422**	100021856|F|0-28:C > A-28:C > A	TGCAGATGGAGACGAGGAGAAGCACGATCGATCCCAGGCCAAAAGGTCCAGCAAATGACATGCAAAGAG
**DART462**	100004133|F|0-16:C > T-16:C > T	TGCAGAGCTCCTCAACCGTGCCTATTTATCTGCACATGGAGCCTTCAGGGCTTCAGGAAAAATCGCATC
**DART4851**	100021552|F|0--65:T > C	TGCAGGCATGATCGGAAGTTTTCCCATGCCGCCTCATTCACATCCCAACCGAAATCAACAACAAATAAG

DArT locus names used throughout manuscript derived from clone ID/SNP variant and associated sequence read.

.

Bioinformatic analysis of the interval containing DArT-seq markers with an *r*^*2*^ value > 0.81 spanned 5 Mb in total and was gene-rich with 75 genes (>60% uncharacterized) (Table 5). The haplotype block analysis using significant DArT-seq markers showed very high linkage disequilibrium among these markers (data not shown). From the 75 predicted transcripts within this region, there was also a relatively large representation of various transcription factors, while only three predicted disease resistance proteins were identified including NB-ARC, NBS-LRR and a serine/threonine receptor kinase (Table 5)

**Table 5:** Bioinformatic analysis of functional annotation based on Pfam of the 75 predicted genes in the genomic region on Morex 5HL at the *RphCantala* locus based on linkage disequilibrium analysis performed on linked DArT-seq markers

**Gene identifyer**	**Physical postion in Morex genome**	**Sequence annotation Pfam**	**Length (amino acid residues)**
MLOC_67435	Chromosome 5: 504,807,504-504,808,889	Uncharacterised protein	1282
MLOC_63880	Chromosome 5: 504,837,984-504,844,416	Uncharacterised protein	4770
MLOC_63879	Chromosome 5: 504,837,985-504,840,518	Peptidase domain	1900
MLOC_14877	Chromosome 5: 504,848,281-504,851,127	Uncharacterised protein	2578
MLOC_72642	Chromosome 5: 504,851,380-504,854,051	Uncharacterised protein/transmembrane	818
MLOC_15752	Chromosome 5: 504,856,869-504,860,258	Uncharacterised protein//serine/threonine protein kinase	1279
MLOC_67813	Chromosome 5: 504,866,847-504,868,082	Uncharacterised protein	156
MLOC_58355	Chromosome 5: 504,868,629-504,870,096	Uncharacterised protein/Myb homeobox	96
MLOC_39379	Chromosome 5: 504,881,559-504,882,690	CytochromeP450/uncharacterised protein	193
MLOC_55782	Chromosome 5: 505,224,634-505,228,648	Uncharacterised protein/Bromodomain	350
MLOC_12507	Chromosome 5: 505,231,551-505,235,061	Transmembrane domain/uncharacterised protein	99
MLOC_56550	Chromosome 5: 505,251,077-505,252,833	Uncharacterised protein	212
MLOC_67579	Chromosome 5: 505,267,327-505,268,018	Uncharacterised protein	73
MLOC_79114	Chromosome 5: 505,304,761-505,305,695	Uncharacterised protein	180
MLOC_38843	Chromosome 5: 505,401,971-505,402,516	Uncharacterised protein/zipper	144
MLOC_22183	Chromosome 5: 505,411,541-505,418,638	Uncharacterised protein	345
MLOC_22184	Chromosome 5: 505,411,592-505,420,058	Microtubule-associated protein	556
MLOC_44070	Chromosome 5: 505,421,803-505,425,401	Uncharacterised protein	346
MLOC_61309	Chromosome 5: 505,435,524-505,436,686	Uncharacterised protein	176
MLOC_67384	Chromosome 5: 505,437,805-505,442,456	Uncharacterised protein/BIPPOZ fold	190
MLOC_14335	Chromosome 5: 505,450,976-505,451,416	Uncharacterised protein	46
MLOC_78241	Chromosome 5: 505,457,197-505,458,593	Glycosyltransferase 2	363
MLOC_37117	Chromosome 5: 505,487,438-505,491,448	Uncharacterised protein/MIP1 Leuczipper	608
MLOC_63425	Chromosome 5: 505,505,232-505,509,647	Uncharacterised/AP2 transcription factor	631
MLOC_58589	Chromosome 5: 505,517,232-505,521,660	Eukaryotic translation initiation factor 3 subunit C	868
MLOC_71335	Chromosome 5: 505,523,834-505,525,773	Cytochrome P450/Uncharacterised protein	453
MLOC_44341	Chromosome 5: 505,572,931-505,576,253	Uncharacterised protein	515
MLOC_11008	Chromosome 5: 505,604,769-505,610,921	Kinesin motor domain/uncharacterised protein	340
MLOC_55124	Chromosome 5: 506,586,998-506,588,618	Ribosomal S14/predicted protein	56
MLOC_55125	Chromosome 5: 506,594,987-506,597,286	Uncharacterised protein	284
MLOC_64140	Chromosome 5: 506,607,831-506,611,618	Malate dehydrogenase	358
MLOC_39143.3	Chromosome 5: 506,613,176-506,622,502	Uncharacterised protein	503
MLOC_4524	Chromosome 5: 506,631,764-506,637,466	Uncharacterised protein	144
MLOC_63065	Chromosome 5: 506,640,602-506,644,654	Uncharacterised protein/protein-kinase domain leucine rich repeat	608
MLOC_37278	Chromosome 5: 506,647,621-506,649,939	Uncharacterised/cytP450	515
MLOC_11920	Chromosome 5: 506,671,388-506,671,569	Uncharacterised protein	
MLOC_61101	Chromosome 5: 506,676,442-506,678,678	Alpha/beta hydrolase domain/uncgharacterised	377
MLOC_52788	Chromosome 5: 506,712,820-506,716,335	Alpha-tubulin 4	451
MLOC_52896.1	Chromosome 5: 506,720,817-506,722,983	Myb/Homeobox/Uncharacterised protein	299
MLOC_77955	Chromosome 5: 506,728,560-506,729,777	Homeobox/leucine zipper/uncharacterised protein	154
MLOC_52360.1	Chromosome 5: 506,734,900-506,736,938	tRNA-butesine synthase/uncharacterised protein	240
MLOC_52361	Chromosome 5: 506,737,533-506,741,133	Pentatricopeptide/uncharacterised	682
MLOC_17956	Chromosome 5: 506,743,784-506,750,717	Uncharacterised protein/N-acetylglucosaminyl transferase component (Gpi1)	541
MLOC_66827	Chromosome 5: 506,758,816-506,764,116	AMP-binding enzyme/uncharacterised protein	700
MLOC_71512	Chromosome 5: 506,765,725-506,767,027	Oligopeptide transporter	292
MLOC_71514	Chromosome 5: 506,771,066-506,774,511	Uncharacterised protein	947
MLOC_36533	Chromosome 5: 506,774,624-506,778,739	Giberellin signalling	548
MLOC_36533	Chromosome 5: 506,779,918-506,782,319	NB-ARC protein/uncharacterised protein	591
MLOC_79498	Chromosome 5: 506,783,900-506,786,958	Peptidase/protein inhibition/Uncharacterised protein	377
MLOC_17927	Chromosome 5: 506,792,732-506,798,457	WRKY transcription factor /Uncharacterised protein	1032
MLOC_66348	Chromosome 5: 506,818,362-506,820,563	NAM protein	337
MLOC_16892	Chromosome 5: 506,827,938-506,828,895	Tetraspanin/peripherin/uncharacterised protein	183
MLOC_14114	Chromosome 5: 506,834,758-506,838,333	FAD reductase/uncharacterised	430
MLOC_23699	Chromosome 5: 506,884,357-506,888,420	RNA polymerase III	120
MLOC_70664	Chromosome 5: 506,907,468-506,911,192	Lipoxygenase	863
MLOC_17894	Chromosome 5: 506,912,481-506,913,432	Uncharacterised protein	145
MLOC_10360	Chromosome 5: 506,913,595-506,916,553	NBS-ARC-LRR disease resistance protein/Uncharacterised	766
MLOC_19228	Chromosome 5: 506,923,278-506,929,445	Cullin repeat-like superfamily	351
MLOC_3679	Chromosome 5: 506,929,610-506,930,486	GTP binding Elongation factor	113
MLOC_10567	Chromosome 5: 506,948,227-506,953,875	Inosine-5′-monophosphate dehydrogenase	496
MLOC_44818	Chromosome 5: 506,995,429-506,995,800	Uncharacterised protein	30
MLOC_76586	Chromosome 5: 506,996,531-506,997,720	Uncharacterised protein	51
MLOC_66071	Chromosome 5: 509,522,514-509,527,272	Calmodulin binding protein/uncharacterised	588
MLOC_66074	Chromosome 5: 509,528,020-509,529,376	Photosystem I reaction center subunit III	439
MLOC_62114	Chromosome 5: 509,534,583-509,540,642	Calmodulin binding protein/uncharacterised	615
MLOC_61300	Chromosome 5: 509,556,146-509,556,624	Uncharacterised protein/Zinc finger	137
MLOC_61302	Chromosome 5: 509,560,844-509,561,551	Uncharacterised protein/Zinc finger	156
MLOC_2917	Chromosome 5: 509,561,748-509,564,141	Protein binding GTP-Elongation Factor	322
MLOC_66343	Chromosome 5: 509,613,233-509,613,914	Uncharacterised protein	59
MLOC_7003	Chromosome 5: 509,663,223-509,664,904	Chaperone J	96
MLOC_4695	Chromosome 5: 509,722,893-509,727,266	F-box domain cyclin/Uncharacterised protein	411
MLOC_66212	Chromosome 5: 509,843,699-509,844,569	Uncharacterised protein	245
MLOC_4789	Chromosome 5: 509,860,275-509,861,218	Uncharacterised protein	146
MLOC_12556	Chromosome 5: 509,866,159-509,866,865	Uncharacterised protein	110
MLOC_66971	Chromosome 5: 509,878,445-509,880,991	Ribose 5P isomerase	

.

## Discussion

Here we report on the discovery and mapping (genetic and physical) of a new seedling resistance allele to *P. hordei*, previously temporarily designated as *RphC.* A previous genetic study using a large F_2_ population determined that the *Rph12* resistance locus in ‘Triumph’ was an allele of *Rph9* [14] and was therefore re-designated as *Rph9.z* based on nomenclature described in Franckowiak et al. [23]. Our studies demonstrated that *RphC* is an allele of *Rph12* (*Rph9.z*) found in ‘Triumph’ based on tests of allelism, chromosomal location and pathotype specificity. Tests of allelism were performed in this study by intercrossing ‘Cantala’ with barley differential lines carrying *Rph3, Rph7, Rph10, Rph12* and *Rph13. RphC* was independent from *Rph3, Rph7, Rph10* and *Rph13* based on observed segregation ratios conforming to two-gene prediction models. Further genetic analysis of F_3_ populations demonstrated that there was no segregation for resistance to *P. hordei* pathotype 253P- between ‘Cantala’ and ‘Triumph’ , suggesting that *RphC* and *Rph12* are likely allelic and therefore *RphC* should be designated *Rph9.am.*

Both multipathotype analysis and observed ITs between ‘Cantala’ , ‘Stirling’ and 13 other Australian barley cultivars postulated to carry *Rph9.am* suggest that the resistance mapped in this study is the same gene. *Rph9.am* had different specificity than *Rph12* (*Rph9.z*). *P. hordei* pathotype 253P- was avirulent on *Rph9.am* and *Rph12*, however pathotypes 210P+ and 200P- were avirulent on *Rph12* yet virulent on *Rph9.am.* Genetic analysis of the CI 9214/Stirling DH population using the 253P- pathotype conformed to a single gene inheritance contributed by the ‘Stirling’ parent. Further assessment of this population with *P. hordei* pts 243P+ (avirulent on *Rph9.am* and virulent on *Rph19*) and 4610P+ (virulent on *Rph12* and *Rph19*) ruled out the involvement of these genes for the resistance observed in the CI 9214/Stirling population.

*Rph9, Rph12* and *Rph9.am* all map to the long arm of chromosome 5H (5HL). Marker-trait analysis performed in this study using 4,500 DArT markers assigned *Rph9.am* to chromosome 5HL in a similar region to both *Rph9* and *Rph12* although there were no comparable markers between genetic maps to accurately assess comparative positions. Comparative genetic analysis between the ‘Bowman’ and CI 9214/Stirling genetic maps identified six markers in common within a 15 cM region of *Rph9.am* and this region co-located to QTL for barley leaf rust resistance in barley on 5HL located in between 126 and 140 cM from cultivar ‘Scarlett’ [24]. Discrepancies were observed between the order and distance of DArT markers between the parental genetic map (CI 9214/Stirling) and the consensus 'Bowman' genetic maps. This is likely to be attributed to the limited number of lines used for genetic map construction and mapping the *Rph9.am* phenotype. Additional file 2 gives the marker haplotype data for each closely linked DArT to *Rph9.am*. A small proportion of markers had missing data and this may further explain the inaccuracy of the marker order and distance observed in the CI 9214/Stirling genetic map. Bioinformatic analysis on sequence reads derived from DArT markers closely linked to *Rph9.am* (*r*^*2*^ > 0.8) suggests that there are four likely gene candidates for the *Rph9.am/Rph12/Rph9* locus within a 5 Mb gene rich region including an NB-ARC, NBS-LRR and two Serine/Threonine receptor kinases. Variation was observed between the ‘Bowman’ and ‘Morex’ genome assemblies for the physical location of two DArT markers (DART7846 and DART3079), both of which showed highest similarity to the same serine/threonine receptor kinase transcript. Such discrepancies are likely to be either due to errors in assembly between the ‘Bowman’ and ‘Morex’ genomes given their recent release or the absence of MLOC_38941 within ‘Bowman’ , which is seedling susceptible to *P. hordei* and lacks any of the characterised *Rph* genes and *Rph9.am* (R. F. Park, unpublished).

High LD among the significant DArT-seq markers in this region, as indicated by haplotype block analysis, suggests that *Rph9.am* could be located to a broad region on chromosome 5HL spanning at least 5 Mb. Given the population size of the F_3_ families used for tests of allelism, it is possible that *Rph12* and *Rph9.am* could be independent closely linked resistance genes separated by a very small physical distance. Bioinformatic analysis of the genes within the physical region believed to carry *Rph9.am* based on LD and the ‘Bowman’ consensus map suggest the presence of two NBS genes. The role of NBS-LRR genes and their involvement in race specific resistance to rusts and other plant pathogens is well documented [25,26] as is their tendency to cluster within grass genomes due to selection for duplication events in response to evolutionary pressure [27,28]. Alternatively, there is a possibility that the same one of the serine/threonine receptor kinase genes is responsible for resistance to multiple pathotypes of *P. hordei* based on previously reported broad spectrum resistance conferred by both *Rpg1* and *Rpg5* to stem rust in barley. Both *Rpg1* and *Rpg5* encode serine/threonine receptor kinases and are believed to show marked homology. *Rpg5* maps approximately 20 Mb away from the predicted *Rph9.am* locus, however, a recent study screening Australian barley cultivars with the *Rpg5* marker identified only 6 out of the 14 lines postulated to carry *Rph9.am* also carried *Rpg5* suggesting they are not the same gene [29].

The Australian barley cultivars postulated to carry *Rph9.am* were all closely related based on available pedigree information. Multipathotype testing performed in this study using five *P. hordei* pathotypes suggests that *Rph9.am* is likely to be present in 14 Australian cultivars. Further pedigree analysis of all Australian cultivars postulated to carry *Rph9.am* demonstrated strong relatedness and shared ancestral pedigrees through ‘Gull’ and ‘Binder’, which trace the source of the *Rph9.am* resistance to a landrace from either Sweden or the former Czechoslovakia. Both ‘Cantala’ and ‘Stirling’ share a common pedigree in ‘Maja’ that was produced from an intercross between ‘Gull’ and ‘Binder’ [30]. A previous study reporting on the genealogical analysis and diversity of spring barleys released from the former Czechoslovakia and the Czech Republic determined that three ancestral landraces, including ‘LV-Gotland’ , contributed significantly to feed barley cultivars. These cultivars were enriched in germplasm of more productive genotypes and were donors of biotic stress resistance at the sacrifice of malting qualities during breeding [31]. Previous research has also found however that ‘Gull’ is susceptible to Australian *P. hordei* pathotypes and only carried the *Rph4.d* allele at the *Rph4* locus. This evidence suggests that 'Binder' not 'Gull' is the more probable source of the *Rph9.am* resistance from ‘Cantala’.

## Conclusions

Consolidating both previous and present studies, at least three alleles contributing to leaf rust resistance [*Rph9, Rph12 (Rph9.z)* and *Rph9.am* (*Rph9.am*)], each with distinct race specificity, map to chromosome 5HL at potentially the same locus. Of these three alleles, *Rph12* and *Rph9.am* appear to be most common in Australian germplasm. Genetic mapping and LD analysis in this study determined that *Rph9.am* is likely to be located in a physical region spanning 5 Mb on chromosome 5HL. Furthermore, this region contained three potential gene candidates which will inform future gene cloning efforts of the *Rph12/Rph9.am* locus for diagnostic marker development.

### Availability of supporting data

All the supporting data in the manuscript are included as additional files.

## Additional files

Additional file 1:
**Linkage disequilibrium (LD) mapping of DArT-seq markers with respect to the** ***RphCantala*** **binary trait marker. Vertical axis represents the correlation co-efficient values.** The peaks above minimum threshold of 0.5 can be considered as associated with *RphCantala*. The colours blue and red were used to differentiate between chromosomes (1H-7H). Additional file 2:
**Haplotypic data for each DArT marker in linkage disequilibrium (r** ^**2**^ **> 0.8) with** ***RphC*** **.** Missing marker data is denoted by an asterix *.A and B denote the presence and absence of the DArT marker allele respectively.
