# Supplementary figures and images for: Genetic mapping of a new race specific resistance allele effective to Puccinia hordei at the Rph9/Rph12 locus on chromosome 5HL in barley
Source: BMC Plant Biol. 2014 Dec 20;14:1598. doi: 10.1186/s12870-014-0382-4 (PMC4302584; doi:10.1186/s12870-014-0382-4)

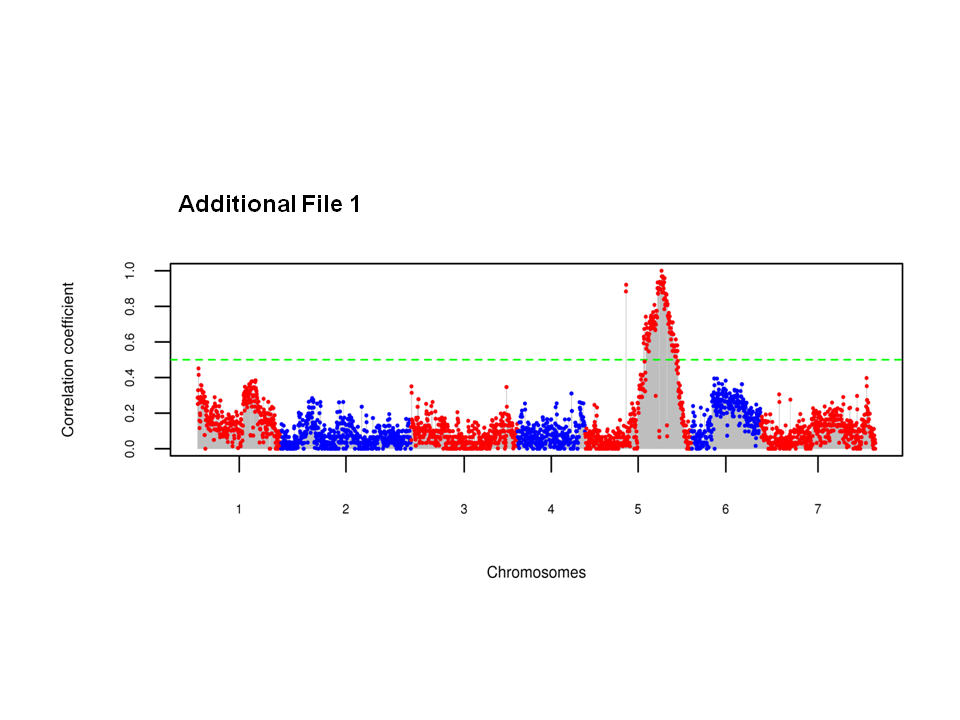

Supplement: Additional file 1: — Linkage disequilibrium (LD) mapping of DArT-seq markers with respect to the RphCantala binary trait marker. Vertical axis represents the correlation co-efficient values. The peaks above minimum threshold of 0.5 can be considered as associated with RphCantala. The colours blue and red were used to differentiate between chromosomes (1H-7H). [file 12870_2014_382_MOESM1_ESM.tiff]
