# Supplementary material for: Genetic mapping of a new race specific resistance allele effective to Puccinia hordei at the Rph9/Rph12 locus on chromosome 5HL in barley
Source: BMC Plant Biol. 2014 Dec 20;14:1598. doi: 10.1186/s12870-014-0382-4 (PMC4302584; doi:10.1186/s12870-014-0382-4)
Supplement: Additional file 2: — Haplotypic data for each DArT marker in linkage disequilibrium (r 2 > 0.8) with RphC . Missing marker data is denoted by an asterix *.A and B denote the presence and absence of the DArT marker allele respectively. [file 12870_2014_382_MOESM2_ESM.doc]

**Additonal File 2**

CloneID DH-1 DH-105 DH-109 DH-11 DH-114 DH-116 DH-123 DH-127 DH-129 DH-130 DH-132 DH-15 DH-153 DH-16 DH-160 DH-166 DH-171 DH-178 DH-179 DH-181 DH-184 DH-185 DH-188 DH-19 DH-195 DH-196 DH-198 DH-20 DH-201 DH-217 DH-225 DH-228 DH-23 DH-234 DH-248 DH-27 DH-273 DH-279 DH-3 DH-37 DH-38 DH-4 DH-42 DH-43 DH-47 DH-49 DH-5 DH-50 DH-6 DH-60 DH-61 DH-65 DH-66 DH-7 DH-74 DH-8 DH-85 DH-89 DH-9 DH-91 DH-94 Stirling CI9214

DART4872 100025017|F|0--28:G>C A B B B A A A A B B B A A B A A B * A A A A B A A B A B A A A * B A A B B B A B B A A B A A B * B B B B B A A A B B A B B A B

DART7508 100020485|F|0--40:G>A * B B * A A A A B B B A A * A A B * A A A * B A B B A B A A A A B A A * B B A B B A A B A A B A B B B B * A * A B B A B B * B

DART7846 100023795|F|0--13:G>A A B B * A A A A B B B A A B A A * * A A A * B A * A * B * * * A * A A * * B * B * A A B A A * * * * B B B A A A * * A * B A B

DART3080 100017949|F|0--37:A>G A B B B A A A A B B B A A B A A B B A A A * B A A B A B A A A A B A A B B B A B B A A B A A B A B B B B B A A A B B A B B A B

DART3263 100023711|F|0--33:C>A B A A A B B B B A A A B B A B B A A B B B B A B A A B A B B B B A B A A A A B A A B B A B B A B A A A A A B B B A A B A A B A

DART4851 100021552 B A A A B B B B A A A B B A B B A A B B B B A B A A B A B B B B A B A A A A B A A B B A B B A B A A A A A B B B A A B A A B A

DART5481 100009066|F|0--42:A>G B A A A * B * B A A A B * A * * A A * B B * A B A A * A B B B * A B A A A A * A A B B A B B A * A A A A A B B * A A B A A * A

DART214 100002081|F|0--29:C>A A B B B A A A A B B B A A B A A B B A A A A B A A B A B A A A A B A A A B B A B B A A B A A B A B B B B B A A A B B A B B A B

DART2133 100025595|F|0--17:C>A B A A A B B B B A A A B B A B B A A B B B B A B A A B A B B B B A B A A A B B A A B B A B B A B A A A A A B B B A A B A A B A

DART2681 100009568|F|0--11:A>G A B B B A A A A B B B A A B A A B B A A A A B A A B A B A A A A B A A B B B A B B A A B A A B A B B B B B A A A B B A B B B B

DART6198 100012213|F|0--56:T>C B A A A B B B B A A A B B A B B A A B A B B A B A A B A B B B B A B A A A B B A A B B A B B A B A A A A A B B B A A B A A * A

DART4228 100017110|F|0--68:A>G A B B B A A A A B B B A A B A A B B A A A A B A A B A B A * A A B A A B B A A B B A A B A A B A B * B B B A A * B B A B B A B

DART4182 100011760|F|0--59:G>A B A A A B B B B A A A B B * B B A A B A B B A B A A A A B B B B A * B A A B B A A B B A B B A B A * A A A B B * A A B A A B A

DART5422 100021856|F|0--28:C>A A B * B A A * A * B B A A B A A B * A B A * B A B B A B A * A A B A * B B A A B B A A B * A B A B B B B B A A A * B A B B A B

DART462 100004133|F|0--16:C>T A B B B A A A A B B B A A B A A B B A B A A B A A B A B A A A A B A A B B A A B B A A B A A B A B B B B B A A A B B A B B A B

RphC A B B B A A A A B B B A A B A A B B A A A A B A B B A B A A A A B A A B B B A B B A A B A A B A B B B B B A A A B B A B B A B
